# Chitosan-Grafted Graphene Oxide-Reinforced Bio-Based Waterborne Epoxy Nanocomposites for Antibacterial and Corrosion Resistance in Tropical Marine Environments: A Mini-Review

**DOI:** 10.3390/polym17212964

**Published:** 2025-11-06

**Authors:** Yunyang Wu, Zhongyuan Luo, Yucheng Wang, Chengwei Xu, Yuanzhe Li

**Affiliations:** 1 Carbon Neutrality Institute, China University of Mining and Technology, Xuzhou 221116, China; 2 College of Polymer Science and Engineering, Sichuan University, Chengdu 610065, China

**Keywords:** bio-based epoxy, chitosan–graphene oxide, antibacterial coating, corrosion resistance, tropical environment, water-based paint, sustainability

## Abstract

Epoxy resin coatings are widely employed for steel protection owing to their excellent adhesion, chemical stability, mechanical strength, and barrier properties. However, conventional bisphenol A-based resins and organic solvents may pose risks to reproductive, developmental, and immune systems, as well as contribute to atmospheric pollution. This mini-review critically evaluates recent advancements in fully waterborne bio-based epoxy nanocomposites as sustainable alternatives, with particular emphasis on their enhanced antibacterial and corrosion-resistant performance in tropical marine environments. A central focus is the role of chitosan-grafted graphene oxide (Chi-GO) as a multifunctional nanofiller that significantly enhances both antibacterial efficacy and barrier capabilities. For instance, coatings reinforced with Chi-GO exhibit up to two orders of magnitude lower corrosion current density than pristine epoxy coatings, and achieve over 95% bacterial inhibition against *Escherichia coli* and *Staphylococcus aureus* at a 1 wt.% loading. The review summarizes key synthesis methods, functional modification techniques, and commonly adopted evaluation approaches. Emerging research further underscores environmental performance metrics, including reduced volatile organic compound (VOC) emissions and improved life-cycle assessments. By integrating bio-based polymer matrices with Chi-GO, these composite systems present a promising pathway toward environmentally benign and durable protective coatings. Nevertheless, critical challenges concerning scalability and long-term stability under real-world operating conditions remain insufficiently addressed. Future research should emphasize scalable manufacturing strategies, such as roll-to-roll processing, and conduct extended tropical exposure testing (e.g., salt spray tests beyond 2000 h). Additionally, developing comprehensive life-cycle assessment (LCA) frameworks will be crucial for sustainable industrial implementation.

## 1. Introduction

Steel, particularly carbon steel, remains a fundamental material in modern infrastructure and industrial applications owing to its advantageous strength-to-cost ratio [1]. Nevertheless, its inherent susceptibility to corrosion significantly compromises long-term durability, particularly in chloride-rich marine environments. The porous rust layer formed during corrosion facilities water and oxygen penetration, accelerating structural degradation and raising substantial safety and economic concerns [2,3]. According to NACE International reports (2016), global economic losses due to corrosion amount to approximately 3–4% of GDP annually, highlighting the urgent need for effective protective strategies. Protective coatings represent the most practical and cost-effective approach to mitigate steel corrosion, with ideal systems providing not only an effective barrier against corrosive agents but also sustained functionality following mechanical or chemical damage. Conventional bisphenol A (BPA)-based epoxy coatings are widely recognized for their strong adhesion, chemical resistance, and barrier properties. However, their reliance on petroleum-derived precursors and organic solvents raises environmental and health concerns, including volatile organic compound (VOC) emissions and endocrine-disrupting effects. Regulatory frameworks such as REACH have increasingly restricted the use of BPA due to its toxicity, driving the search for safer alternatives. Waterborne polyurethane (WPU) coatings have emerged as promising substitutes, offering improved safety, adhesion, and flexibility [4,5]. However, their comparatively lower crosslinking density and hydrophilic nature often result in inadequate corrosion resistance in saline environments. This limits their applicability in demanding settings such as tropical marine systems [6]. Furthermore, once damaged, conventional coatings often require labor-intensive repairs, reducing efficiency in large-scale marine applications.

In response, bio-inspired and sustainable coating technologies have garnered significant attention. Innovations include self-healing coatings that mimic biological repair mechanisms to autonomously restore integrity, as well as bio-based epoxy resins derived from renewable resources. Despite progress, achieving concurrent high durability, long-term stability, and robust corrosion resistance under tropical marine conditions continues to be a major challenge. The incorporation of nanofillers has shown considerable promise in overcoming these limitations. Among these, graphene oxide (GO) nanosheets offer exceptional barrier properties, mechanical strength, and chemical stability, enabling the development of composite coatings with enhanced performance [7,8]. Functionalized derivatives like Chi-GO further enhance dispersibility and interfacial bonding, while also imparting antibacterial functionality—an essential feature for coatings in microbe-rich tropical marine environments. This mini-review critically examines recent advances in fully waterborne, bio-based epoxy nanocomposites reinforced with Chi-GO, with a specific focus on their enhanced antibacterial and corrosion-resistant properties in tropical marine environments. We highlight the unique role of Chi-GO as a multifunctional nanofiller that synergistically improves barrier properties and antimicrobial efficacy. The core contribution of this work lies in its focused analysis of Chi-GO modified aqueous bio-based epoxy systems, addressing both antibacterial and anti-corrosion mechanisms within the context of tropical marine service environments—a perspective underexplored in existing literature.

## 2. Review of Waterborne Bio-Based Epoxies

### 2.1. Raw Material Types and Synthesis Methods of Bio-Based Epoxy Resins

Bio-based epoxy resins represent a promising sustainable alternative to conventional petroleum-derived epoxies, addressing concerns over toxicity, non-renewability, and environmental impact. Current research focuses on renewable feedstocks such as lignocellulosic biomass, food-processing byproducts, and vegetable oils. Lignin, a major constituent of wood, exhibits a phenolic structure analogous to bisphenol A (BPA), making it a viable candidate for epoxy synthesis [9]. It can be extracted via sulfonation, organic solvent, or hydrolysis methods, and further functionalized through epoxidation [10], depolymerization, or glycosylation [11] to enhance reactivity and compatibility. Aromatic compounds derived from food waste—such as cardanol, vanillin, furan derivatives, and eugenol—also show potential as epoxy monomers or curing agents. Meanwhile, vegetable oils including epoxidized soybean oil (ESO), epoxidized linseed oil (ELO), and epoxidized castor oil (ECO) are rich in unsaturated bonds that can be epoxidized to introduce epoxy functionalities, often yielding flexible resin formulations. Table 1 provides a comprehensive overview of different properties of conventional and bio-based epoxy resins.

Although these bio-based epoxies can match or exceed the mechanical properties of their petroleum-based counterparts in some aspects, they frequently require blending or hybridization to achieve balanced performance [15,16]. Typical synthesis routes involve direct epoxidation, reaction with epichlorohydrin (ECH) to form glycidyl esters, or cross-linking with bio-derived curing agents [17]. It is noteworthy that a bio-content of at least 20% is generally required to confer significant environmental benefits; however, thermomechanical properties—such as glass transition temperature (Tg) and tensile strength—often decline with increasing bio-content, underscoring the need for performance optimization through structural design, copolymerization, or nanocomposite reinforcement [18].

### 2.2. Advantages and Challenges of Waterborne Epoxy Systems

Waterborne epoxy resins (WERs), which use water as a dispersion medium, offer significant advantages in reducing volatile organic compound (VOC) emissions and promoting environmental and operational safety. They retain the strong adhesion, chemical resistance, and barrier properties of solvent-based systems, making them suitable for marine corrosive environments. Ease of cleanup and reduced maintenance further enhance their practical value. However, WERs typically exhibit slower curing kinetics and greater sensitivity to temperature and humidity compared to solvent-borne systems, which can lead to inferior film formation. The presence of water often results in porous microstructures, reducing sealing capability and water resistance. Additionally, WERs generally demonstrate lower initial adhesion, impact resistance, and chemical stability, along with increased susceptibility to cathodic disbondment, blistering, and premature failure in highly humid or saline environments [18]. Issues such as storage stability, sedimentation, and freeze–thaw resistance also require continual improvement [19].

While the incorporation of bio-based epoxies—either as nanomaterials or toughening agents—can partially mitigate these drawbacks by enhancing mechanical and anti-corrosion performance, the long-term durability of waterborne bio-based epoxies under harsh conditions (e.g., high humidity, temperature, and UV exposure) remains inadequately validated. Most existing studies remain confined to laboratory-scale formulations, lacking long-term performance data in real marine service conditions. Moreover, when bio-content exceeds 30%, a significant decline in mechanical properties is often observed, necessitating the use of nanoreinforcements such as chitosan-grafted graphene oxide (Chi-GO) to restore performance, as summarized in Table 2.

## 3. Introduction and Functional Modification of Nano-Fillers in Epoxy Composites

### 3.1. Fundamentals and Limitations of Graphene Oxide

**Figure 1:**
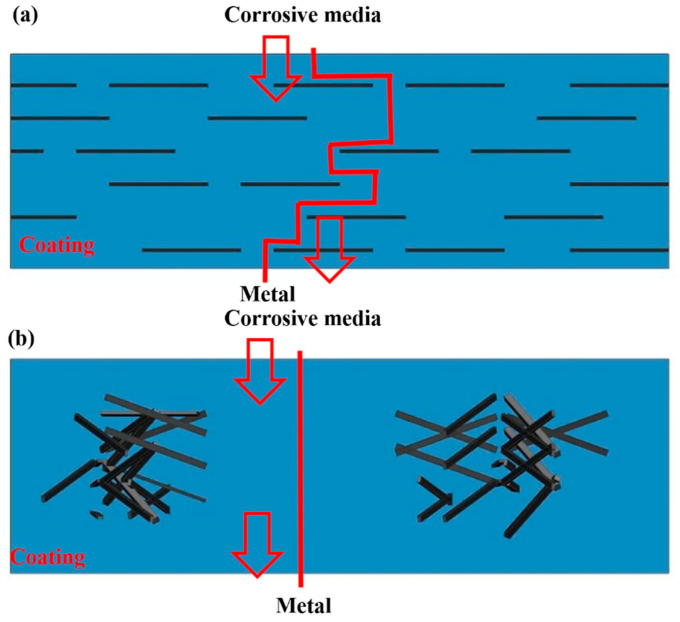
The maze effect of GO in epoxy resin. (**a**) Well-dispersed graphene prolonging the permeation path of the corrosive materials and (**b**) poorly dispersed coating showing a short permeation path [33]. Copyright 2019 Gui G., et al.

Graphene oxide (GO) has garnered significant attention as a nano-filler in epoxy composites due to its exceptional barrier properties, high specific surface area, and abundant oxygen-containing functional groups (e.g., hydroxyl, epoxide, carboxyl) [30]. The “maze effect” is widely recognized as the primary mechanism for its barrier performance: impermeable GO nanosheets dispersed within the polymer matrix create highly tortuous pathways, drastically prolonging the diffusion distance of corrosive species such as H_2_O, O_2_, and Cl^−^, thereby delaying the onset and progression of corrosion (Figure 1) [31]. Meanwhile, the high specific surface area of GO, which is close to the theoretical value of 2630 m^2^/g, endows it with a large number of active sites of oxygen-containing functional groups, which can be combined with organic matrices such as polyaniline and epoxy resin or inorganic particles such as α-Fe_2_O_3_/Fe_3_O_4_ and SiO_2_ through covalent/non-covalent interactions, reducing the defect rate and hindering the passage of corrosive ions significantly lowers the corrosion current density of the coating [32].

Despite these advantages, GO suffers from inherent limitations. Its strong interlayer π–π interactions and van der Waals forces lead to agglomeration, resulting in poor dispersion within the polymer matrix and the formation of defect sites that compromise composite performance [33,34]. Moreover, In the absence of specific functionalization or post-synthesis modification, GO tends to exhibit limited antimicrobial efficacy and may even serve as a scaffold that facilitates initial microbial attachment at sub-microgram-per-milliliter to low tens of microgram-per-milliliter concentrations [35].

### 3.2. Functionalization Strategies and Mechanisms of Chitosan-Grafted GO

To overcome these limitations, functionalization strategies have been developed, among which grafting chitosan (CS) onto GO has proven highly effective. CS, a bio-based, biodegradable, and non-toxic polysaccharide, is rich in amino and hydroxyl groups. It can be covalently bonded to carboxyl groups on GO via carbodiimide-mediated amidation. In the MES(2-(N-Morpholino)ethanesulfonic acid) buffer solution (with a pH of 5), EDC(1-Ethyl-3-(3-dimethylaminopropyl)carbodiimide) and NHS(N-hydroxysuccinimide) were used as coupling agents to activate the carboxyl groups on the surface of GO, allowing them to react with the amino groups on the chitosan chains to form amide bonds. Thus, the GO-CS nanocomposite material was successfully synthesized. The formation of amide bonds was confirmed by FTIR and XPS [36]. In addition, Chitosan-grafted GO can also be synthesized through the electrostatic assembly method. This method disperses graphene oxide (GO) in water, then adds acetic acid (HAc) to adjust the pH and protonate the amino groups in chitosan (CS) to (–NH_3_^+^). Subsequently, it self-assembles with negatively charged GO through electrostatic interactions to form a physically cross-linked network structure. This non-covalent interaction (including hydrogen bonds and electrostatic forces) is the main driving force for the formation of the hydrogel, and it achieves effective composites of CS and GO under acidic conditions, endowing the hydrogel with pH responsiveness, enhanced mechanical properties and self-healing ability [37].

Chitosan-Grafted GO can enhance the performance of graphene oxide through three mechanisms.

- **Improved Dispersion**

Chitosan (CS) grafting effectively reduces the interfacial tension and enhances the compatibility between graphene oxide (GO) and the epoxy matrix by leveraging strong hydrogen bonding and electrostatic interactions [38]. This improved interfacial affinity promotes more uniform exfoliation and homogeneous distribution of GO nanosheets, thereby minimizing agglomeration-induced defects and forming a continuous, tortuous barrier network within the composite.

- **Antibacterial Functionality**

The protonated amino groups of CS under acidic conditions enable strong electrostatic adsorption onto negatively charged bacterial membranes, causing membrane disruption, content leakage, and cell death [39]. This significantly mitigates MIC, with studies reporting higher inhibition against *E. coli* and *S. aureus* [40].

- **Enhanced Interfacial Adhesion**

The introduction of CS improves the interfacial bonding between GO and the epoxy matrix, facilitating efficient stress transfer and resulting in superior mechanical properties (e.g., tensile strength, fracture toughness) and corrosion resistance [41]. Electrochemical impedance spectroscopy (EIS) measurements show that Chi-GO-modified coatings exhibit impedance modulus values higher than pure epoxy [42].

### 3.3. Comparison and Synergy with Other Nano-Fillers

Various nano-fillers have been explored for enhancing epoxy coatings, each with distinct advantages and limitations. Table 3 below summarizes the key performance metrics of GO, Chi-GO, and other common nano-fillers. The Chi-GO hybrid material demonstrates synergistic multifunctionality, offering concurrent antimicrobial and anti-corrosion properties. The chitosan component provides robust, broad-spectrum antibacterial activity that effectively inhibits microbiologically influenced corrosion (MIC), while the well-dispersed GO nanosheets significantly enhance the coating’s barrier resistance and passivation effects against electrochemical corrosion. In contrast, conventional nanofillers such as CNTs and SiO_2_ primarily offer single-function enhancement: CNTs improve electrical conductivity and mechanical reinforcement, and SiO_2_ augments hardness and pore-sealing capability, but neither intrinsically possesses antimicrobial activity, thereby limiting their effectiveness in environments requiring dual protective functions.

The assessment of “Antibacterial Activity” in Table 3 is primarily based on the efficacy against common marine bacteria and the prevention of biofilm formation, which is crucial for mitigating MIC. Chitosan (CS) possesses inherent antibacterial properties due to the interaction of its positively charged amino groups with the negatively charged bacterial cell membranes, leading to cell lysis and death [43]. Graphene oxide (GO) can also exhibit antibacterial effects through physical disruption but is generally considered weaker than CS alone. The Chi-GO hybrid synergistically combines these mechanisms, often resulting in superior (>95% inhibition) and broad-spectrum antibacterial performance, as extensively documented [44]. In contrast, CNTs and SiO_2_ typically show negligible intrinsic antibacterial activity unless specifically functionalized [45].

The “Sustainability” metric evaluates the environmental impact of the nano-filler, considering factors such as the renewability of the raw material source, energy consumption during production, and biodegradability. Cellulose nanocrystals (CNC) and Chi-GO score highly due to their derivation from abundant, renewable biopolymers (cellulose and chitin, respectively). Their production processes are generally less energy-intensive compared to synthetic alternatives, and they offer better prospects for biodegradation or biocompatibility [46,47]. GO and SiO_2_ have moderate sustainability profiles; GO can be produced from graphite, a natural resource, but often involves harsh chemical oxidation steps, while SiO_2_ is abundant but its processing can be energy-intensive. CNTs, typically synthesized from fossil fuels via high-energy processes like chemical vapor deposition, rank lowest in terms of green credentials and renewability [48].

Apart from the type of nano-fillers, the content also significantly affects the corrosion resistance of the coating. Studies have shown that the carbon nanotube with a loading of up to 7 wt.% in the epoxy matrix was desirable for corrosion resistance [49]. On the other hand, graphene contents of up to 1 wt.% were desirable to enhance the corrosion resistance of the epoxy matrix [49]. Carburized epoxy coating with 6% modified silica cured was more corrosion resistant in nature [50]. As a comparison, when the loading amount of Chi-GO is 1 wt% [51], it shows excellent corrosion resistance, with the surface corrosion degree being only 5% [51], while for GO with the same loading amount, the surface corrosion degree reaches 15% [51].

Chitosan-grafted graphene oxide (Chi-GO) represents a highly promising multifunctional nano-filler for epoxy composites, effectively addressing the dispersion challenges of GO while imparting significant antibacterial and corrosion-inhibiting properties. Its ability to enhance both mechanical performance and environmental sustainability positions it as a superior candidate for protective coatings in aggressive environments. However, the long-term stability of Chi-GO under high salinity, elevated temperature, and prolonged exposure remains inadequately validated, necessitating further research into its durability under real-world tropical marine conditions.

## 4. Comparative and Critical Analysis of the Antibacterial and Corrosion-Resistant Mechanisms of Chitosan, Graphene Oxide, and Chitosan-Grafted Graphene Oxide

### 4.1. The Antibacterial and Corrosion-Resistant Mechanisms of Chitosan

#### 4.1.1. Electrostatic Adsorption and Membrane Disruption

**Figure 2:**
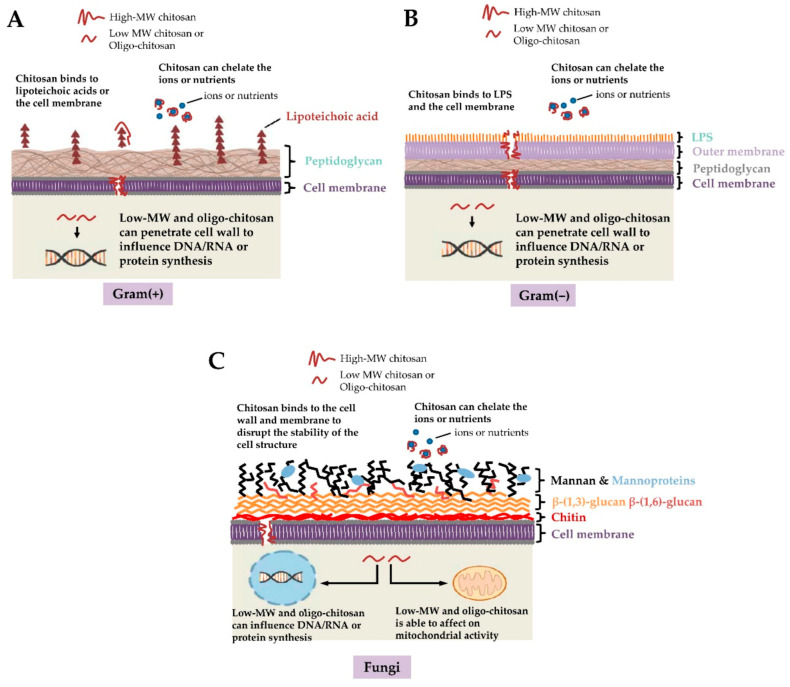
Potential antimicrobial actions of chitosan against (**A**) Gram-positive bacteria, (**B**) Gram-negative bacteria, and (**C**) fungi. Given the negative charges of teichoic acids in Gram-positive bacteria, lipopolysaccharide (LPS) in Gram-negative bacteria and the phosphorylated mannosyl side in fungi, electrostatic interactions occur between the positively charged chitosan and the cell surface of the microorganism. Furthermore, chitosan chelates the environmental ions and nutrients required for bacterial survival. Low-molecular weight (MW) chitosan and oligo-chitosan might affect DNA/RNA or protein synthesis after passing through the cell wall and cell membrane into the cytoplasm. Additionally, low-MW chitosan and oligo-chitosan inhibit mitochondrial function and ATP production [56]. Copyright 2021 Ke C. et al.

Chitosan, as a natural cationic polysaccharide, exhibits antibacterial activity mainly due to its unique polycationic nature. Under acidic conditions, the free amino groups in chitosan molecules undergo protonation, forming positively charged –NH_3_^+^ groups, which strongly adsorb onto the bacterial cell membrane surface through electrostatic interactions [1]. The bacterial cell membrane usually has a negative charge, such as the phosphocholine (teichoic acid) in the cell wall of Gram-positive bacteria (e.g., *Bacillus cereus*) (Figure 2), which contains a large number of phosphate groups and has a strong negative charge, thus having a higher affinity for chitosan. This adsorption effect can disrupt the integrity of the cell membrane, cause depolarization of the membrane potential and disruption of the transmembrane ionic gradient, and further lead to increased membrane permeability. The loss of the cell membrane barrier function allows potassium ions, ATP, proteins, and other biological macromolecules to leak out of the cell, while abnormal intracellular substances flow in, ultimately triggering cell apoptosis or necrosis. Additionally, chitosan may enter the cell and bind to DNA or RNA [52], interfering with the replication and transcription of genetic material, thereby enhancing its antibacterial effect. Multiple studies have indicated that the antibacterial efficacy of chitosan is closely related to its molecular weight [53], deacetylation degree, concentration [54], and environmental pH value [55]. Chitosan with high deacetylation degree and medium molecular weight usually exhibits stronger antibacterial effects under acidic conditions. This mechanism is not only applicable to Gram-positive bacteria but also has a certain inhibitory effect on Gram-negative bacteria (such as *Escherichia coli*), but the intensity of its effect varies due to differences in cell wall structure and composition.

#### 4.1.2. Induction of Oxidative Stress

Secondly, chitosan can also trigger oxidative stress by inducing the mitochondrial pathway and accumulating endogenous reactive oxygen species (ROS), thereby exerting its antibacterial effect. Multiple studies have confirmed that chitosan can penetrate the bacterial cell membrane and interfere with the electron transport chain, promoting the massive generation of ROS such as superoxide anion, hydrogen peroxide, and hydroxyl radicals (˙OH) [57]. When the ROS level exceeds the clearance capacity of the bacterial antioxidant defense system (such as superoxide dismutase, catalase, etc.), it will cause severe oxidative damage, including lipid peroxidation of the cell membrane, protein carbonization and denaturation, and DNA strand breaks. This irreversible molecular damage further leads to metabolic dysfunction and may activate the conserved apoptotic-like cell death pathway within the bacteria (such as caspase-like proteinase activation) [58]. Different from the physical destruction mechanism that directly acts on the cell membrane, the ROS-mediated antibacterial pathway is more dependent on biochemical processes, and it still maintains good activity in the presence of certain resistant bacterial strains (such as methicillin-resistant *Staphylococcus aureus*, MRSA), demonstrating its potential application value in combating multi-drug-resistant microorganisms. Therefore, chitosan, as a multi-mechanism synergistic antibacterial agent, its ability to induce oxidative stress expands its application prospects in biomedicine and preservation protection.

#### 4.1.3. Concentration-Dependent Efficacy

The antibacterial activity of chitosan is highly concentration dependent. Higher concentrations enhance electrostatic interactions, physical barrier formation, and ROS induction. Studies report a transition from bacteriostatic to bactericidal effects at specific concentrations [59]. Efficacy is also influenced by molecular weight and degree of deacetylation, with medium–low molecular weight and high deacetylation degree generally yielding superior activity.

#### 4.1.4. Corrosion Resistance Mechanism

The corrosion resistance of chitosan is manifested as a combined protective mechanism of both active and passive nature. On one hand, CS can form a dense film on the metal surface, providing a physical barrier to prevent the penetration of corrosive media; on the other hand, the abundant amino and hydroxyl functional groups in its molecules can effectively chelate metal ions, promoting the formation of a stable passivation layer at the interface and inhibiting the dissolution of the anode metal. Moreover, CS has certain pH buffering capacity for the local corrosion microenvironment, which can slow down the formation of acidic microregions. However, the strong hydrophilicity of CS and its relatively weak mechanical strength limit its application as a long-term anti-corrosion coating alone. Usually, it needs to be compounded with other resins or nanomaterials to make up for these deficiencies.

### 4.2. The Antibacterial and Corrosion-Resistant Mechanisms of Graphene Oxide

GO, with its high specific surface area and two-dimensional layered structure, effectively prolongs the diffusion paths of corrosive media such as water, oxygen and chloride ions through the “maze effect”, significantly enhancing the physical barrier performance of the coating. However, the antibacterial performance of GO is relatively weak, and even at low concentrations, it may promote bacterial adhesion and biofilm formation, exacerbating microbial-induced corrosion (MIC). Its problems of easy aggregation and poor dispersibility also limit its practical application in composite coatings. The corrosion resistance of GO mainly relies on its physical barrier ability, lacking the chemical function of actively inhibiting corrosion.

### 4.3. The Synergistic Enhancement Mechanism of Chitosan-Grafted Graphene Oxide

The integration of CS and GO yields a composite (Chi-GO) with enhanced antibacterial performance through:

- **Enhanced Electrostatic Adsorption**

Chitosan (CS) introduces a high density of protonated amino groups (–NH_3_^+^) onto the surface of the nanocomposite, creating a strong positive charge under acidic or neutral conditions. This cationic characteristic significantly enhances electrostatic adhesion to the negatively charged components of bacterial cell membranes, such as lipopolysaccharides in Gram-negative bacteria and teichoic acids in Gram-positive bacteria. The improved adsorption efficiency not only increases the local concentration of antibacterial agents around the bacterial cells but also promotes subsequent physical interactions—including membrane disruption and potential leakage of intracellular components—thereby amplifying the overall bactericidal efficacy.

- **Carrier Effect**

Graphene oxide (GO) serves as an excellent nanocarrier platform for chitosan (CS), significantly enhancing its functional performance through several key mechanisms. The exceptionally high specific surface area and abundant oxygen-containing functional groups (e.g., –COOH, –OH) of GO provide numerous active sites for the covalent immobilization or strong physical adsorption of CS molecules, substantially increasing CS loading capacity compared to conventional carriers. This combination not only improves the dispersion stability of CS in aqueous environments, preventing its aggregation, but also enables a more controlled and localized release profile. The hybrid nanostructure ensures that CS is delivered and retained at the target interface (e.g., a coating surface), enhancing its sustained antibacterial efficacy while mitigating premature diffusion or deactivation.

- **Multi-Mechanistic Action**

The Chi-GO (Chitosan–graphene Oxide) nanocomposite exhibits a potent multi-mechanistic antibacterial action that overcomes the limitations of its individual components. This synergistic effect integrates three primary bactericidal pathways: (1) the cationic chitosan (CS) molecules electrostatically adsorb onto negatively charged bacterial cell membranes, disrupting membrane integrity and inducing permeability and leakage of intracellular contents; (2) the sharp, two-dimensional edges of the well-dispersed GO nanosheets exert physical “nanoknife” effects. It explains that the sharp, two-dimensional edges of well-dispersed GO nanosheets function like “nanoknives.” Upon making close contact with a cell, these rigid and ultra-thin edges mechanically puncture, cut, and tear the cell envelope. This direct physical damage compromises the cell’s structural integrity, leading to the leakage of cellular contents and ultimately causing cell death; and (3) both components, particularly GO under certain conditions, can facilitate the generation of reactive oxygen species (ROS), causing oxidative stress that damages cellular components such as proteins, lipids, and DNA.

- **Optimized Composition**

**Figure 3:**
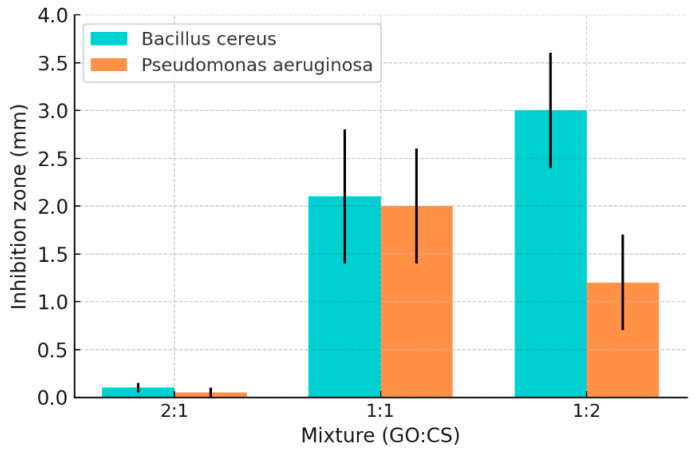
Inhibition zone measurement by BC and PAO1 at different ratios of GO: CS composite [60]. Copyright 2022 Ashry.

In terms of antibacterial performance, the composite system shows a significant proportion-dependent effect (Figure 3): when GO:CS = 2:1, due to the higher proportion of GO, its negative charge neutralizes the positive charge of CS, thereby weakening the antibacterial effect of CS and even promoting bacterial growth; while when the ratio is adjusted to 1:2, the composite shows the optimal antibacterial performance, with significantly higher inhibition diameters and cell viability loss rates for both Gram-positive bacteria (such as *Bacillus cereus*) and Gram-negative bacteria (such as *Pseudomonas aeruginosa*) compared to a single component.

From the perspective of corrosion resistance, GO provides a physical barrier, while CS enhances interface compatibility and introduces an active inhibition function. In a local acidic corrosion environment, CS protonates to form a cation-selective interface that repels Cl^−^ and promotes the formation of a metal passivation layer. In addition, Chi-GO has a photothermal response self-repairing ability, which can rapidly repair microcracks under near-infrared light and restore the integrity of the coating.

### 4.4. Performance Comparison and Case Analysis

Table 4 conducts a comparative analysis of the advantages and disadvantages of GO and CS in terms of antibacterial and corrosion resistance and summarizes the advantages of Chi-GO.

Table 5 [61] summarizes comparative antibacterial performance of CS, GO, and Chi-GO against common and marine strains [60]. Recent studies (2020–2024) demonstrate that Chi-GO-reinforced epoxy coatings maintain >90% antibacterial efficiency against marine bacterial strains after 7 days of immersion in artificial seawater, underscoring their potential for long-term service.

### 4.5. Critical Analysis of Current Research

Despite the promising multifunctionality of chitosan-functionalized graphene oxide (Chi-GO) nanocomposites, several significant limitations and research gaps remain to be addressed before their widespread application can be realized.

- **pH Sensitivity**

The antimicrobial efficacy of chitosan (CS) is highly dependent on its protonation state. In neutral or alkaline environments (pH ≥ 7), the amino groups (–NH_2_) on CS remain deprotonated, drastically diminishing its cationic charge density and, consequently, its electrostatic adsorption capacity to negatively charged bacterial cell walls. This inherent pH sensitivity severely limits the standalone application of Chi-GO in environments where the pH is not acidic, such as in marine coatings or biological fluids.

- **GO Stability and Performance Degradation**

A critical challenge lies in the long-term colloidal stability of GO nanosheets. In high-salinity environments (e.g., seawater), the elevated ionic strength induces electrostatic shielding effects, leading to rapid aggregation, sedimentation, and potential leaching of GO from the composite matrix. This aggregation not only diminishes the nanocarrier function and specific surface area of GO but also compromises its physical “nanoknife” effect, ultimately resulting in a significant decline in barrier and antimicrobial performance over time.

- **Ecotoxicological Uncertainty**

The long-term environmental fate and ecotoxicological impact of Chi-GO nanocomposites represent a substantial research gap. While the individual components are often touted as “green,” the potential synergistic toxicity of their nanohybrid form is not well-understood. There is a pressing lack of comprehensive life-cycle assessment studies evaluating the persistence, bioaccumulation, and toxicity of these nanomaterials towards non-target aquatic organisms and soil microbiota, hindering their environmentally responsible deployment.

- **Future Directions**

To overcome these limitations, future research should be directed along several strategic pathways. The development of multi-stimuli-responsive smart release systems is paramount. Designing Chi-GO hybrids that can respond to environmental triggers such as local pH changes, elevated temperature, or enzymatic activity at the infection/corrosion site would enable a targeted, on-demand release of antimicrobial agents, maximize efficacy while minimize unnecessary environmental release. Furthermore, integration with other green antimicrobial agents could create next-generation synergistic hybrids. Incorporating natural extracts, antimicrobial peptides, or bio-based metal–organic frameworks (MOFs) into the Chi-GO architecture could offset the pH limitations of chitosan and provide a broader spectrum of action with reduced risk of resistance development.

## 5. Corrosion Resistance Enhancement by Chi-GO: Mechanisms, Evaluation and Challenges

### 5.1. Corrosion Challenges in Tropical Marine Environments

**Figure 4:**
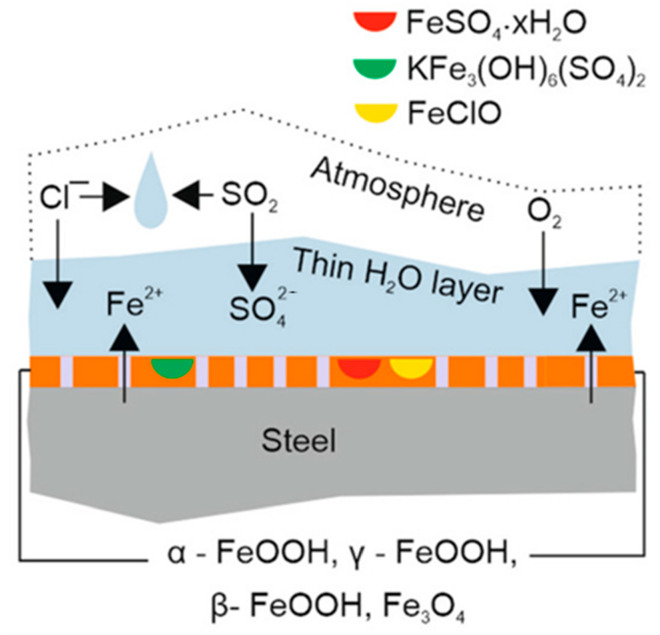
The tropical marine environment accelerates the corrosion of steel [64]. Copyright 2022 Seechurn Y. et al.

The tropical marine environment is recognized as one of the most corrosive environments for metal materials and protective coatings due to the combined effects of high salt spray, high temperature and humidity, and strong ultraviolet radiation (Figure 4). In this region, the deposition rate of chloride ions can reach several hundred milligrams per square meter per day, effectively destroying the passive film on the metal surface and significantly accelerating the electrochemical corrosion process. Meanwhile, the continuous high temperature (annual average temperature of 27.5 °C) and high humidity (RH > 80%) promote the diffusion of reactants and electrochemical processes. Strong ultraviolet radiation (annual radiation amount of about 7000 MJ/m^2^) can also induce photo-oxidative degradation of organic coatings, leading to powdering and loss of adhesion. To simulate these harsh conditions, international standards such as ISO 12944-2:2018 [62], the protective performance of coating systems for C5-M marine environments is evaluated through accelerated cyclic tests simulating harsh conditions, such as salt spray, UV exposure, and humidity; ASTM B117 [63] stipulates the use of a 5% NaCl solution, a constant temperature of 35 °C and a salt spray deposition rate of 1.0–2.0 mL/80 cm^2^/h for accelerated tests. It should be noted that the multi-factor coupling effects in actual environments (such as the synergistic effect of NH_4_^+^ and Cl^−^ in triggering “quasi-autocatalytic pitting” on magnesium alloys) far exceed the single conditions in the laboratory. Therefore, a comprehensive assessment should be made by combining real sea exposure data with standards such as ISO 12944-2:2018 [62].

### 5.2. Chi-GO Electrochemical Testing Technology and Application Cases

- **Electrochemical Impedance Spectroscopy (EIS)**

Electrochemical impedance spectroscopy (EIS) is a non-destructive quantitative method used to evaluate the barrier performance and durability of coatings in a 3.5 wt.% NaCl solution. The tests are typically conducted at 25 °C with a frequency range of 10 mHz to 100 kHz and an amplitude of 50 mV. A three-electrode system is employed, with Ag/AgCl as the reference electrode, platinum as the counter electrode, and the coated Q235 carbon steel as the working electrode [65]. Through the analysis of Nyquist and Bode plots, key parameters such as coating capacitance (Qc), coating resistance (Rc), and charge transfer resistance (Rct) can be obtained, which reflect the evolution trend of the coating’s shielding performance. The Chi-GO modified coating exhibits significantly enhanced performance: compared with the pure epoxy coating, the charge transfer resistance (Rct) increases by 5 to 10 times, and the low-frequency impedance modulus (|Z|0.01 Hz) reaches 10^8^ Ω·cm^2^ (an increase of two orders of magnitude). These data indicate that the Chi-GO coating has excellent barrier durability and interface inhibition ability.

- **Scanning Vibrating Electrode Technique (SVET)**

The scanning vibrating electrode technique (SVET) enables spatially resolved monitoring of the local electrochemical activity in the defect areas of coatings through micro-area current density mapping and is particularly suitable for evaluating the self-healing behavior and local corrosion inhibition effect of coatings. In this technique, a vibrating microelectrode scans the defect area of the coating to measure the local current density distribution, thereby identifying the regions and intensity changes in anodic dissolution and cathodic oxygen reduction reactions. For the Chi-GO coating, SVET analysis shows that the anodic current density is significantly suppressed at the artificial scratch, confirming effective corrosion protection. This suppression effect is significantly enhanced under near-infrared (NIR) light, as the photothermal trigger promotes the rapid release of chitosan inhibitors and barrier recovery, leading to nearly complete restoration of the damaged area and uniform current distribution, reflecting the intact coating performance. The study indicates that under NIR irradiation, the anodic current density in the scratch area of the Chi-GO coating is reduced by approximately 80%, while the control coating only shows a slight self-healing effect.

- **Tafel Polarization**

Tafel polarization analysis provides crucial quantitative insights into the corrosion kinetics and thermodynamic trends of coated substrates. For the chitosan–graphene oxide (Chi-GO) modified coating, this technique consistently shows a significant positive shift in the corrosion potential (Ecorr), indicating enhanced thermodynamic stability and a reduced driving force for the anodic metal dissolution reaction. More importantly, a systematic observation reveals a reduction in the corrosion current density (Icorr) by one to two orders of magnitude, clearly demonstrating a substantial decrease in the corrosion rate. This suppression of Icorr is directly attributed to the synergistic barrier effect of well-dispersed graphene oxide nanosheets, which prolongs the diffusion path, along with the active inhibition provided by chitosan molecules released at defect sites. Specific data show that the Icorr value of the Chi-GO coating drops to approximately 10^−9^ A/cm^2^, while that of the pure epoxy coating is about 10^−7^ A/cm^2^ as indicated in Table 6 [66], confirming the excellent anti-corrosion performance of the Chi-GO coating.

### 5.3. Summary of the Mechanism of Nanofillers in Enhancing Corrosion Resistance

Chitosan–graphene oxide (Chi-GO) nanocomposites significantly enhance the corrosion resistance of epoxy coatings through four synergistic mechanisms. Table 7 shows the characteristics of the four mechanisms.

#### 5.3.1. Physical Barrier Effect

The physical barrier effect is the cornerstone of the anti-corrosion mechanism of graphene oxide (GO) nanosheets in polymer coatings. When properly dispersed, the high aspect ratio two-dimensional GO nanosheets align parallel to the substrate, forming a highly tortuous path that significantly prolongs and hinders the diffusion of corrosive substances such as water molecules, oxygen, and chloride ions towards the metal substrate [67]. The incorporation of chitosan (CS) maximizes this “maze” effect. Research shows that after being modified by organosilicon and nano-diamond, the GO coating remains an impedance modulus of 1.00 × 10^9^ Ω·cm^2^ after being immersed in a 3.5 wt.% NaCl solution for 120 days, which is three orders of magnitude higher than that of the pure resin coating, with a protection efficiency as high as 97.8% [68]. The key to the “palace effect” lies in CS, which acts as a green dispersant and compatibilizer. This is an optimized and enhanced version of the “maze effect”. As a green dispersant, CS interacts with the GO sheets through its functional groups, reducing the van der Waals forces between the GO sheets and effectively preventing the aggregation and restacking of GO. A more perfect barrier network is formed: after GO is better exfoliated and dispersed, it can form a more continuous, dense and defect-free sheet network in the epoxy resin matrix. By utilizing its functional groups, it reduces the van der Waals forces between GO sheets, minimizing restacking and agglomeration, thereby achieving more uniform exfoliation and superior interfacial compatibility with the epoxy resin matrix. In terms of chemical interaction and interface enhancement, the abundant amino and hydroxyl groups in chitosan form strong covalent bonds and dense hydrogen bond networks with the carboxylic acid functional groups on the GO nanosheets, significantly enhancing interfacial adhesion, minimizing phase separation, and promoting effective stress transfer, thereby suppressing the nucleation and propagation of microcracks. Regarding interface regulation, the protonated amino groups of CS impart a cationic nature to the modified GO, significantly improving its compatibility and interfacial adhesion with the epoxy resin matrix. At the onset of corrosion, local acidification further protonates it, forming a cation-selective interface that electrostatically repels chloride ions and may promote the formation of a protective layer through chelation of metal cations. In terms of functional self-healing, the integration of Chi-GO introduces advanced functional self-repairing capabilities, mainly mediated by its photothermal responsiveness under near-infrared irradiation. The photothermal conversion efficiency of GO enables rapid local heating, enhancing the fluidity of chitosan chains and dynamically altering the hydrogen bond network, thereby quickly sealing microcracks and achieving complete defect closure within 30 s, restoring the barrier integrity of the coating.

#### 5.3.2. Chemical Interactions and Interface Enhancements

The reinforcing effect of chitosan–graphene oxide (Chi-GO) modified epoxy coatings stems from the multiple chemical interactions at the interfaces among its components. The abundant amino groups (–NH_2_) and hydroxyl groups (–OH) in chitosan (CS) molecules undergo amidation reactions with the carboxyl groups (–COOH) on graphene oxide (GO) nanosheets, forming stable covalent bonds. Meanwhile, a dense hydrogen bond network is established among CS chains, GO nanosheets and the epoxy resin matrix, significantly enhancing the interfacial bonding strength [69]. These strong interfacial interactions increase the interfacial bonding strength of the composite material by approximately 50% and effectively suppress the phase separation phenomenon.

#### 5.3.3. Interface Regulation

In terms of interface regulation, the protonated amino groups (–NH_3_^+^) of CS endow the modified GO with significant cationic properties, greatly enhancing its dispersion and interfacial compatibility in polar epoxy resins. Research shows that the uniformity of GO nanosheets dispersion in Chi-GO composite coatings is approximately 40% higher than that of unmodified GO, significantly reducing coating defects [70]. More importantly, this cationic property exhibits an intelligent response function at the initial stage of corrosion: the local acidification in the anode area promotes further protonation of CS, forming a cation-selective interface that effectively repels corrosive anions such as Cl^−^ (reducing the corrosion rate by about 60%), while promoting the formation of a protective metal chelate layer, providing an additional active protective mechanism for the coating.

#### 5.3.4. Self-Healing Function

Self-healing ability is a prominent feature of the Chi-GO system. The excellent photothermal conversion efficiency of GO (up to ~45%) enables rapid local heating under near-infrared (NIR) irradiation. This thermal effect activates a dual repair mechanism: on the one hand, it enhances the fluidity of CS chains, promoting their migration to the microcrack areas; on the other hand, it dynamically reconstructs the hydrogen bond network to achieve rapid sealing of the damaged areas [71]. Experiments show that the Chi-GO coating can completely close microcracks within 30 s under NIR irradiation, with a repair efficiency of over 92%, far exceeding that of traditional self-healing coatings (which usually take several hours to several days). This rapid and efficient self-healing property significantly extends the service life of the coating and provides continuous and reliable corrosion protection for metal substrates.

### 5.4. Engineering Challenges and Research Gaps

Although Chi-GO nanocomposites exhibit outstanding anti-corrosion performance, their engineering application still faces multiple challenges. Insufficient durability is one of the key issues. Long-term exposure to ultraviolet radiation and high temperatures can lead to the photo-oxidative degradation of the polymer matrix and chitosan components, reducing the interfacial adhesion and compromising the structural integrity of the coating. Research indicates that under continuous thermal cycling conditions, the differential expansion and contraction between the organic and inorganic phases promote the accumulation of internal stress, causing micro-cracking, delamination, and relaxation of the aligned GO nanosheets, thereby weakening the maze effect. Performance evaluation under complex coupled environments is still insufficient. The actual marine environment involves the simultaneous action of salt spray, temperature cycling, and microbial communities, while current studies mostly remain at the single-factor experimental level, lacking performance data under multi-factor coupled conditions. The lack of standardization severely restricts the development and certification of smart coatings. Traditional testing standards (such as ASTM B117 [63] and ISO 12944 [62]) mainly focus on passive barrier performance and fail to quantitatively assess self-healing kinetics, trigger antibacterial efficacy, and performance degradation under multi-factor coupled conditions (such as simultaneous UV irradiation, thermal cycling, and microbial activity), which hinders reliable comparison and validation of smart coatings. Ecological safety has not been evaluated, and the long-term toxicological impact of GO and its derivatives in marine ecosystems remains inadequately studied. Systematic assessment is urgently needed for the environmental fate, persistence, bioaccumulation potential, and toxicological effects of these nanofillers on non-target marine organisms at different trophic levels. Special attention should be paid to the transformation pathways of released nanoparticles under UV and saltwater conditions, their interaction with organic matter, and their sub-lethal effects on marine microbial communities, invertebrates, and fish. The complexity of Chi-GO hybrids introduces additional variables, and in-depth research is needed on the degradation products of chitosan and their synergistic effects with GO.

These research gaps highlight the limitations of current technologies: Firstly, accelerated aging test methods fail to fully simulate the multi-factor coupling conditions of the real environment, resulting in a gap between laboratory data and field performance. Secondly, material characterization techniques have insufficient resolution in real-time monitoring of the self-healing process of coatings and the degradation mechanism at interfaces. Thirdly, the lack of standardized testing protocols in ecotoxicological studies makes it difficult to assess the impact of nanomaterials on the marine environment throughout their life cycle. Finally, the absence of predictive models hinders the accurate assessment of coating life and the optimal design of material performance.

Chitosan-grafted graphene oxide, as a multifunctional nanofiller, shows great potential for application in tropical marine environments by synergistically enhancing the antibacterial and anticorrosive properties of waterborne bio-based epoxy coatings. Its core advantage lies in providing a triple protection mechanism of physical barrier, chemical interface enhancement, and biological inhibition, offering a compelling strategy for the next generation of protective coatings. Research indicates that GO-based coatings, after appropriate modification, maintain an impedance value above 10^8^ Ω·cm^2^ after being immersed in a 3.5 wt.% NaCl solution for 100 days, which is two orders of magnitude higher than that of pure epoxy coatings [72]. However, to achieve its full application, interdisciplinary collaboration (integrating materials science, microbiology, and corrosion engineering) is necessary to address issues of scalability, long-term durability under actual multi-stress conditions, and ecological safety. Future research should focus on promising directions such as developing multi-stimuli-responsive coatings (pH, photothermal, and electrochemical synergy), establishing predictive life models to accurately assess the service performance of coatings in complex environments, and integrating life cycle assessment (LCA) into material design to ensure the sustainable and safe deployment of these advanced materials in marine ecosystems. Special attention should be paid to the dual functions of Chi-GO hybrid materials in tropical marine multi-factor corrosion environments, which require systematic verification through interdisciplinary approaches, including molecular dynamics simulation of interface interactions, advanced characterization techniques to analyze degradation mechanisms, and standardized ecotoxicological tests to assess environmental risks. Only through these comprehensive methods can such intelligent protective coatings be transformed from laboratory research to engineering applications, providing reliable solutions to metal corrosion problems in marine environments.

## 6. Environmental Sustainability and Industrial Application Prospects

### 6.1. Environmental Friendliness and Sustainability Analysis

The shift towards waterborne bio-based epoxy resins marks a significant step in reducing the environmental footprint of protective coatings [73]. Compared to conventional solvent-based epoxy systems, which are derived from petroleum and emit high levels of volatile organic compounds (VOCs), waterborne bio-based epoxies significantly reduce VOC emissions (by 60–80%) and utilize renewable resources such as lignin and vegetable oils, thereby diminishing reliance on toxic precursors like bisphenol A (BPA).

Beyond the resin matrix itself, the choice of nanofiller plays a crucial role in determining the overall environmental impact of the nanocomposite. Carbon Nanotubes rank low in terms of sustainability and renewability [74]. Their potential environmental persistence and ecotoxicity also raise concerns. While SiO_2_ is abundant in nature, its processing into nano-forms can be energy-intensive. It generally exhibits moderate sustainability but lacks inherent biodegradability [75]. On the other hand, GO can be produced from graphite, a natural resource. However, its conventional synthesis often involves harsh chemical oxidation methods that use hazardous reagents and generate waste [60], posing potential risks of VOC residue and environmental contamination if not managed properly. This results in a moderate sustainability rating. In contrast, Chi-GO stands out as a more environmentally friendly option. Its chitosan component is derived from chitin, an abundant, renewable, and biodegradable biopolymer sourced from seafood industry waste. The grafting of chitosan onto GO can mitigate some of the dispersion and aggregation issues associated with pristine GO, potentially leading to more durable coatings and reduced nanomaterial leaching. Furthermore, the use of Chi-GO imparts antibacterial functionality without relying on heavy metals or synthetic biocides, aligning with greener coating philosophies. Consequently, Chi-GO is recognized as a high-sustainability nanofiller due to its bio-based origin and the potential for reduced environmental impact throughout its life cycle.

However, comprehensive environmental, health and safety (EHS) assessments, particularly for nano-components like GO and Chi-GO, are still lacking [76]. The long-term ecological impact and potential synergistic toxicity of these nanohybrids in marine environments require systematic evaluation. Future work should prioritize greener synthesis routes for nanomaterials, such as utilizing agricultural waste to produce GO and Chi-GO, to avoid organic solvents entirely. Furthermore, the adoption of life cycle assessment (LCA) methodologies is crucial for conducting a full-chain environmental impact evaluation—from raw material acquisition to production, use, and disposal—providing scientific and data-driven support for the sustainable application of these advanced polymer functional coatings.

### 6.2. Current Situation and Challenges in Industrial Applications

At present, the industrial application of high-performance protective coatings is still dominated by traditional solvent-based epoxy and polyurethane systems. Although regulatory pressure and environmental concerns have prompted major manufacturers (such as AkzoNobel, Amsterdam, Netherlands and PPG Industries, Pittsburgh, PA, USA) to invest in the development of water-based and biogenic alternative products, the integration of nano-enhancers like GO and Chi-GO into such systems is mostly still at the laboratory or pilot-scale stage. The main challenges include the dispersion of nano-materials, the feasibility of process scaling-up, and cost-effectiveness.

The industrialization of Chi-GO enhanced bio-based epoxy coatings faces several obstacles such as high cost of nanomaterials, complex functionalization processes, and the lack of standardized quality assurance and performance verification schemes. Although the water-based system reduces the cost of solvents and environmental compliance burdens, the introduction of GO and Chi-GO increases the cost of raw materials. Currently, the chemical oxidation synthesis method for GO has high energy consumption and involves hazardous reagents, while the functionalization with chitosan requires additional steps (such as carbon diimide-mediated grafting), increasing the complexity and cost of the process. Moreover, achieving uniform dispersion and directional arrangement of nanosheets in large-scale coating applications (such as roll-to-roll coating, spraying) remains a technical challenge.

Despite the challenges, relevant explorations have emerged in the industry. For instance, some companies have attempted to introduce graphene-based additives into commercial coating systems, mostly focusing on cement-based or solvent-based systems rather than water-based epoxy. In the maritime and marine engineering field, bio-based epoxy resins based on natural fillers are being evaluated according to standards such as ASTM D5894 [77] (cyclic ultraviolet/condensation test) and ISO 20340 [78] (marine protective coatings). Some pioneering research (such as DSM and BASF) has explored combinations of epoxy resin from oxidized vegetable oil with nanoclay and cellulose nanofibers, demonstrating improved sustainability features and compliance with EU regulations (such as REACH, EU eco-label). However, the application of Chi-GO composite materials is still limited by the lack of standardized testing methods for nano-composite coatings and the absence of long-term field data in tropical marine conditions.

In the future, the industrial application prospects of Chi-GO enhanced bio-based epoxy coatings will depend on whether economic and technical barriers can be overcome through innovations in nanomaterial production, functionalization, and application processes. Strategies such as in situ grafting, continuous flow reactors, and the use of waste biomass to produce graphene are expected to reduce costs and enhance sustainability. Moreover, aligning with international standards (such as developing ASTM/ISO guidelines for nano-reinforced coatings) and strengthening cooperation between industry, academia, and research will be crucial for verifying performance, ensuring compliance, and promoting market acceptance. With the continuous advancement of technology, these multifunctional coatings have broad application prospects in fields such as offshore wind power, marine infrastructure, and chemical processing plants, which have extremely high requirements for durability, antibacterial properties, and environmental compliance.

## 7. Conclusions

This mini-review systematically investigates the development and performance of chitosan-grafted graphene oxide (Chi-GO) reinforced bio-based waterborne epoxy nanocomposites, emphasizing their dual functionality—antibacterial and corrosion-resistant properties—under tropical marine conditions. The incorporation of Chi-GO as a multifunctional nanofiller effectively addresses critical limitations of conventional epoxy systems, including environmental toxicity, poor dispersibility of GO, and the absence of active antimicrobial action.

Key findings demonstrate that Chi-GO significantly enhances coating performance through synergistic mechanisms: The GO nanosheets provide a physical barrier that prolongs the diffusion of corrosive species, while chitosan contributes antibacterial activity via membrane disruption and oxidative stress induction. Electrochemical analyses confirm that Chi-GO-modified coatings exhibit corrosion current densities reduced by up to two orders of magnitude and impedance values exceeding 10^7^ Ω·cm^2^. Furthermore, the composite demonstrates pH-responsive and photothermal self-healing capabilities, further extending service life in aggressive environments.

Nonetheless, challenges remain regarding scalability, long-term durability under real-world multi-stress conditions, and the ecological safety of nanomaterials. Future efforts should prioritize scalable synthesis routes, standardized performance evaluation under multi-factor coupling conditions, and comprehensive life-cycle assessments to ensure sustainable implementation. By integrating bio-based matrices with intelligent nanofillers like Chi-GO, this work provides a viable pathway toward eco-friendly, durable marine protective coatings, bridging material innovation with environmental stewardship.

## Figures and Tables

**Table 1:** Comparative Properties of Conventional and Bio-based Epoxy Resins.

Property	BPA-Based Epoxy	Lignin-Based Epoxy (Lignin Content of 66%)	Vegetable-Oil-Based Epoxy (Niepa Seed Oil/Epoxy (20/80))
Tg (°C)	93 [12]	79 [13]	53.9 [14]
Tensile Strength (MPa)	47.5 ± 3.2 [12]	66 [13]	9.6 ± 0.4 [14]
Corrosion Resistance	High	Moderate	Low to Moderate
VOC Emissions	High	Low	Very Low

**Table 2:** Assessment of Benefits and Limitations of Waterborne Epoxy Systems with Focus on Chitosan-Grafted Graphene Oxide (Chi-GO) Reinforced Bio-Based Epoxy Nanocomposites.

Benefits	Detailed Assessment	Limitations	Detailed Assessment
**Low VOCs & Eco-friendly**	Bio-based epoxy/Chi-GO system reduces petroleum use and VOC emissions, enhancing biodegradability and biocompatibility [20].	**Slow Curing & Humidity Sensitivity**	Hydrophilicity of waterborne/bio-based systems delays curing under humid conditions; Chi-GO partially mitigates water uptake [21].
**Enhanced Barrier & Corrosion Protection**	Chi-GO induces a tortuous diffusion path, with GO–chitosan synergy improving barrier and corrosion resistance in saline environments [22].	**Porous Microstructure & Water Uptake**	Despite Chi-GO, water loss causes micro-porosity, and high humidity retains water, weakening initial barrier function [23].
**Improved Mechanical & Adhesion Properties**	Chi-GO enhances tensile strength, modulus, and metal adhesion via interfacial interactions between chitosan amines and the epoxy matrix [24].	**Reduced Impact Resistance at High Bio-Content**	When bio-content exceeds 30%, toughness decreases. Chi-GO helps recover stiffness but may reduce ductility if dispersion is poor [25].
**Antibacterial Activity**	Chitosan exhibits intrinsic antibacterial activity, reducing microbial-induced corrosion in marine settings; graphene oxide enhances this via membrane disruption [26].	**Durability under UV/Thermal Exposure**	Bio-based epoxies and chitosan degrade under UV and hydrolysis [27], limiting long-term performance in tropical climates without stabilizers.
**Applicability on Damp Surfaces**	Waterborne systems show better damp-substrate tolerance; Chi-GO enhances wet adhesion via chitosan’s hydrophilicity [28].	**Storage & Freeze-Thaw Stability**	Nano-reinforced systems may aggregate after repeated freeze-thaw cycles, impacting shelf life and necessitating optimized dispersion [29].

**Table 3:** Performance Comparison of Nano-Fillers in Epoxy Coatings.

Nano-Filler	Dispersion	Mechanical Enhancement	EIS (Ω·cm^2^)	Antibacterial Activity	Sustainability
GO	Moderate	Moderate	~10^3^–10^4^	Weak	Moderate
Chi-GO	Good	High	~10^7^	Strong (>95% inhibition)	High (Bio-based)
CNT	Poor	High	~10^8^–10^9^	None	Low
CNC	Good	Moderate	~10^5^–10^6^	Moderate	High (Bio-based)
SiO_2_	Good	High	~10^9^	None	Moderate

**Table 4:** Comparative Analysis of GO, CS, and Chi-GO in Terms of Antibacterial and Corrosion Resistance.

Material	Antibacterial Advantage	Antibacterial Limitation	Corrosion Resistance Advantage	Corrosion Resistance Limitations
CS	Broad-spectrum antibacterial, biodegradable	pH sensitivity, poor mechanical properties	Film-forming property, metal chelation	High water affinity, easy dissolution
GO	Physical cutting potential (high concentration)	Easy to reunite, may promote bacterial growth	Extremely strong physical barrier, high specific surface area	No active antibacterial effect, difficult to disperse
Chi-GO	Synergistic antibacterial effects, enhanced pH adaptability		Barrier, Active Inhibition, Self-Repairing Multi-Functional Integration	

**Table 5:** Antibacterial Performance of CS, GO, and Chi-GO Nanocomposites.

Material	Inhibition Rate (%)—*E. coli*	Inhibition Rate (%)—*S. aureus*	MIC (μg/mL)—*Pseudomonas*	Marine Environment Stability
CS	99.99 ± 0.01	99.99 ± 0.01	1000	Poor (pH-dependent)
GO	70	93	>1000	Moderate
Chi-GO (2:1)	>98	>99	200	High

**Table 6:** Comparison of Electrochemical Parameters for Pure WPU, GO-WPU, and Chi-GO-WPU Coating.

Electrochemical Parameter	Pure WPU Coating	GO-WPU Coating	Chi-GO-WPU Composite Coating
Ecorr (V)	−0.224	−0.101	−0.2~−0.6
Icorr (A/cm^2^)	1.91 × 10^−8^	1.74 × 10^−8^	10^−11^~10^−6^
Rp (Ω·cm^2^)	2.30 × 10^6^	2.54 × 10^6^	10^4^~10^7^
|Z|_0.01_ Hz (Ω·cm^2^) (20 days immersion in 3.5 wt.% NaCl solution)	1.36 × 10^7^ → 9.32 × 10^6^	1.49 × 10^7^ → 9.49 × 10^6^	~10^8^

**Table 7:** The characteristics of the four mechanisms.

Mechanism Type	Mode of Action	Performance Manifestation	Key Influencing Factors
Physical Barrier	GO nanosheets form a tortuous maze barrier	Extends the Cl^−^ penetration path by >10 times	GO dispersion, orientation, interlayer spacing
Chemical Interaction	Hydrogen bonding network and dynamic bonding	Enhances interfacial bonding strength, delays cracking	Functional group density, reactivity
Interface Regulation	Charge barrier and adhesion enhancement	Reduces cathodic delamination, improves binding strength	Degree of interfacial chemical reaction
Functional Self-Healing	NIR photothermal response and dynamic bond reorganization	Micro-crack repair efficiency >80%	Stimulus responsiveness, chain segment mobility

## Data Availability

No new data were created or analyzed in this study. Data sharing is not applicable to this article.
